# Tales of a Super Butterfly: Is *Vanessa carye* a Truly Migrant Species? Unraveling Migration Using Morphological and Genomics Approaches

**DOI:** 10.1093/molbev/msaf212

**Published:** 2025-09-19

**Authors:** Amado Villalobos-Leiva, Enrique Rodriguez-Serrano, Franco Cruz-Jofré, Isabel A Lobos, Alejandro Piñeiro González, Javier Pinochet, Hugo A Benítez

**Affiliations:** Laboratorio de Ecología y Morfometría Evolutiva, Centro de Investigación de Estudios Avanzados del Maule, Universidad Católica del Maule, Talca 3466706, Chile; Laboratorio de Mastozoología, Departamento de Zoología, Facultad de Ciencias Naturales y Oceanográficas, Universidad de Concepción, Barrio Universitario S/N, Concepción, Chile; Laboratorio de Mastozoología, Departamento de Zoología, Facultad de Ciencias Naturales y Oceanográficas, Universidad de Concepción, Barrio Universitario S/N, Concepción, Chile; Escuela de Medicina Veterinaria, Facultad de Recursos Naturales y Medicina Veterinaria, Universidad Santo Tomás, Santiago 8370003, Chile; Laboratorio de Ecología y Morfometría Evolutiva, Centro de Investigación de Estudios Avanzados del Maule, Universidad Católica del Maule, Talca 3466706, Chile; Millennium Institute Biodiversity of Antarctic and Sub-Antarctic Ecosystems (BASE), Santiago, Chile; Vicerrectoría de Investigación y Postgrado, Universidad de La Serena, La Serena 1700000, Chile; Departamento de Ecología, Facultad de Ciencias, Universidad Católica de La Santísima Concepción, Concepción, Chile; Laboratorio de Ecología y Morfometría Evolutiva, Centro de Investigación de Estudios Avanzados del Maule, Universidad Católica del Maule, Talca 3466706, Chile; Millennium Institute Biodiversity of Antarctic and Sub-Antarctic Ecosystems (BASE), Santiago, Chile; Instituto One Health, Facultad de Ciencias de la Vida, Universidad Andrés Bello, Santiago, Chile; Cape Horn International Center (CHIC), Centro Universitario Cabo de Hornos, Puerto William, Chile

**Keywords:** migratory behavior, butterfly, movement ecology, genomics, geometric morphometrics

## Abstract

Among movement strategies, migratory behavior is particularly intriguing in insects. Home-breeding is often permanent, and return journeys can take several generations. Although migration is crucial to the ecological and evolutionary processes of the species involved, knowledge of insect migratory behavior needs to be better understood. *Vanessa carye*, a butterfly native to South America with a latitudinal range of ∼7,000 km, exemplifies this problem. This study analyzed samples collected across the species’ range using single-nucleotide polymorphisms (*SNPs*) to assess population structure, genetic diversity, and geometric morphometrics to examine wing shape variation. Results indicate that *V. carye* forms a genetically homogeneous unit composed of only two potential populations spanning ∼5,000 km, geographically correlated with the Pacific Ocean and the Andes, maintaining constant gene flow, and with a mean heterozygosity of 5.74% (SE: ±0.048%). Geometric morphometrics detected no geographic differentiation in wing shapes and sizes across ∼7,000 km, suggesting an absence of local adaptation and indicating a conserved wing shape adapted to flight throughout the species’ range. Our findings support *V. carye* as a migratory species with the longest migratory journey among American butterflies, revealing two migratory routes. With these approaches, we provide a consistent methodological framework for migratory studies in species with important gaps in knowledge of their natural history.

## Introduction

Movement capability is crucial for understanding the ecological and evolutionary processes of species. Among the different types of movements, migratory behavior is characterized as a long-distance seasonal movement at a whole population scale (Dingle et al. 1999; Dingle and Drake 2007; Dingle 2014). It represents a highly costly ecological strategy regarding energy expenditure and increased mortality risk associated with long-distance travel (Alerstam et al. 2003; Dingle 2014). In order to be carried out successfully, it depends on a series of possibly co-evolved physiological and morphological traits, as well as the selection of migratory routes, which are practically immutable through time representing a hereditary trait that defines the populations of the migratory species (O’Corry-Crow et al. 1997; Petit and Mayer 2000; Russell et al. 2005; Dingle and Drake 2007; Lyons et al. 2012; Dingle 2014). Insects and other invertebrates offer a contrasting model compared to iteroparous vertebrates in the study of migratory, as they permanently abandon their natal home ranges and often require several generations to complete a full migratory round trip (Talavera et al. 2018; Menchetti et al. 2019; Chowdhury et al. 2021). This, coupled with insect short lifespan, as well as their high abundance, makes them a suitable model for studying the ecological and population effects of migration processes, as well as for understanding the evolutionary processes involved in this behavior.

Butterflies are interesting species for studying migratory processes, partly because of their charisma and because several species exhibit migratory behavior (Le Roy et al. 2019). Furthermore, the nymphalid genus *Vanessa* stands out, as 6 of its 22 species are considered to exhibit migratory behavior or at least possess high mobility. Then, this trait evolved independently at least 6 times within the genus, where the species considered highly mobile are not closely related (Wahlberg and Rubinoff 2011). One such species is *V. carye*, a medium-sized butterfly native to South America, strongly associated with the Andes Mountains, with a latitudinal range of ∼7,500 km, inhabiting almost the entire continent except for the northernmost eastern part (Peña and Ugarte 1996; Benyamini et al. 2014; GBIF Secretariat 2023). Historically, there has been debate about the migratory status of this butterfly, while other authors consider that it does not have migratory behavior and only has a wide distribution range (Williams et al. 1942; Abbott 1962; Londoño 2018), as it has been considered a species with a great capacity for movement due to its enormous distribution range, as well as the fact that it has been reported in island locations and very distant from the coast of the southeast Pacific (Williams et al. 1942; Abbott 1951; Wahlberg and Rubinoff 2011). More recently, this species was reported to be able to fly above 5,200 meters above sea level (Benítez et al. 2019). Additionally, Escobar-Suárez et al. (2023) reported no evidence of wing morphological differentiation in its altitudinal range of distribution and no evidence of genetic structuring based on mitochondrial molecular markers. These results indicate constant genetic flow between the localities distributed in this altitudinal gradient (Escobar-Suárez et al. 2023). However, its status as a migratory species has not been formally assessed.

Two relevant aspects must be considered when assessing the migratory status of *V. carye*. First, it is essential to evaluate the degree of gene flow and connectivity between populations distributed throughout the species’ range (Monaghan et al. 2001; Claramunt et al. 2012; Alvial et al. 2019; García-Berro et al. 2023; Frayer and Coughlan 2024). In species, weak genetic structure and subtle intraspecific differentiation are often observed, in contrast to nonmigratory or low-dispersal species, which tend to show stronger population divergence and are more prone to speciation (Gaston 1998; Belliure et al. 2000; Helbig 2003; Claramunt et al. 2012; Riginos et al. 2014). For this reason, traditional genetic markers (such as mitochondrial DNA or microsatellites) have not always been effective for studying migratory species (Brattström et al. 2010). However, the use of single-nucleotide polymorphisms (*SNP*s) has improved this limitation, enabling the study of species with weak or absent population structure (Normile 2009; Seeb et al. 2011a; Therkildsen et al. 2013; Chan et al. 2017; Norambuena et al. 2021; Chieu et al. 2023; Nascimento-Schulze et al. 2023). This approach is especially valuable for investigating migratory behavior, as it allows for the analysis of migratory processes over broad geographic scales and discontinuous time periods. Moreover, *SNP*-based methods can aid in the detection of cryptic species within migratory species complexes that alternate between allopatry and sympatry, even in the absence of complete isolation (Battey and Klicka 2017; Chan et al. 2017; Perry et al. 2017).

Second, migratory behavior is a costly strategy requiring specialized adaptations to carry out long-distance trips efficiently. For flying species, a good way to decrease the energy cost during migration is to use wind or air streams to facilitate transportation (Dingle et al. 1999). This implies a specialized wing shape for this purpose. Insects and birds that migrate have larger and narrower wings compared to nonmigratory species, which facilitates the use of air streams, increasing the gliding periods and thus allowing energy savings (Dingle et al. 1999; Dingle 2006; Chapman et al. 2010; Claramunt et al. 2012). In the case of Lepidoptera, Kuussaari et al. (2014) reported a positive relationship between wingspan and dispersal rates, although without assessing migratory behavior between species. Moreover, in the case of species identified as migratory, differences in wing morphology have been observed between populations with different migratory behaviors. For example, in *Danaus plexippus*, it has been observed that individuals from migratory populations have larger and elongated forewings with more acute-angled apices compared to individuals from resident populations (Dockx 2007; Altizer and Davis 2010; Li et al. 2016). Also, differences in wing shape have been detected between populations with migratory routes of different lengths in *D. plexippus* and the dragonfly *Pantala flavescens* (Berns 2014; Alvial et al. 2019). Furthermore, variations in butterfly wing shape have been observed in response to habitat differences, with notable differences even occurring over a few meters along a vertical distribution gradient (Chazot et al. 2016; Dell’Aglio et al. 2022). This suggests that wing shape is a valuable trait for detecting local adaptations, as distinct wing shapes would be expected under pronounced environmental gradients (Le Roy et al. 2019). Overall, migratory species tend to exhibit larger and more elongated wings compared to sedentary species, as these traits enhance flight efficiency over long distances (Dockx 2007; Berns 2014; Alvial et al. 2019).

In this study, we used *SNPs* and geometric morphometrics (GM) to analyze the population structure of *V. carye* across its distribution range. Our first goal was to determine whether this species forms a single panmictic population, consists of multiple genetically distinct populations, or represents a cryptic species complex. Additionally, we aimed to assess whether *V. carye* exhibits a consistent wing shape adapted for flight across its entire range or if there is sufficient variation to suggest local adaptations linked to independent ecological units.

## Materials and Methods

### Geographic Sampling

**Fig. 1.**
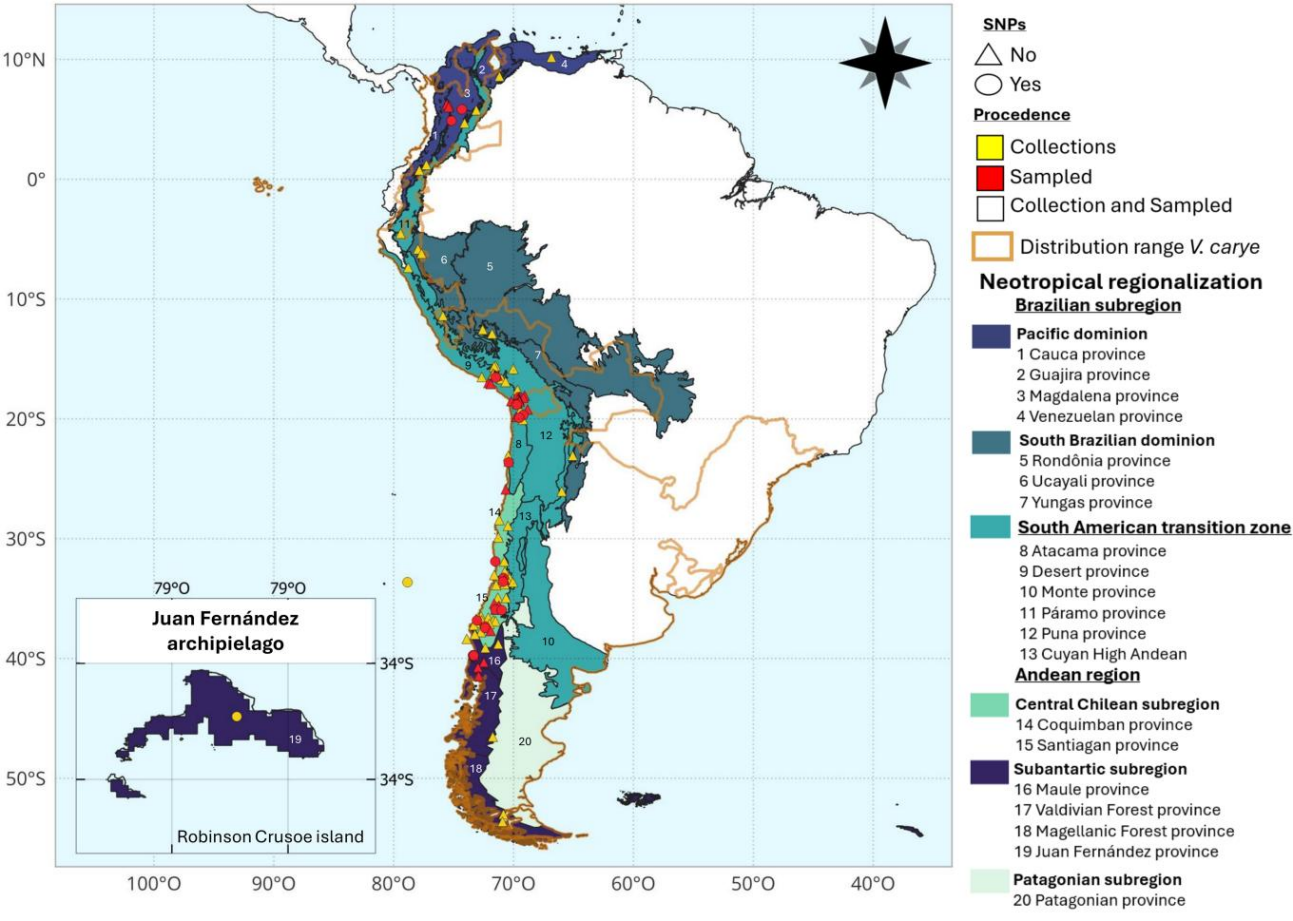
Map of sample locations across the distribution range of *Vanessa carye*. Sampling sites are represented by points, where colors indicate sample provenance (collections, field sampling, or both), and shapes denote the availability of genomic data (circle = yes, triangle = no). The orange outline represents the described distribution range of the species (GBIF Secretariat 2023). Additionally, sampling localities are classified according to Neotropical biogeographic regions and provinces, following the framework proposed by Morrone (2018) and Morrone et al. (2022).

A total of 489 individuals from 56 localities were collected during fieldwork conducted between April 2018 and April 2023, in addition to 500 individuals from 71 localities obtained from private collections (supplementary table S1, Supplementary Material online). In total, 989 individuals were analyzed from 125 localities distributed between 8°N and 53°S, covering the entire latitudinal range of the species’ distribution (Fig. 1).

For field-collected samples, each individual was georeferenced according to its sampling locality with a GPS Garmin GPSMAP 64csx. Wings were extracted and mounted on glass plates following their physiognomic scheme to facilitate their photography and digitization. The bodies were preserved in 90% ethanol and stored at −20 °C. Individuals from private collections were only used for morphological analysis, as no tissue was extracted for genetic studies. The only exception was the Juan Fernández Archipelago samples, from which genetic material was obtained due to their low DNA degradation, using the same protocols as field-collected samples.

Wings were classified according to their integrity, and only those in which the wing structures could be fully identified were included in the geometric morphometric analyses. Damaged wings were excluded from the present analyses, but they were classified according to their type of damage and stored for potential use in future studies.

A total of 1,774 wing samples were obtained for GM analyses, comprising 907 forewings and 866 hindwings from 124 localities. Meanwhile, due to budgetary constraints and sequencing costs, only individuals with high-quality DNA (i.e. low fragmentation) were selected for genomic analysis, prioritizing a minimum of 5 samples per locality whenever possible. As a result, 140 individuals were sent for sequencing from 21 localities.

To ensure biogeographically meaningful classifications, all sampling localities were assigned to ecoregions following Morrone et al. (2022) and further refined to Andean provinces based on Morrone (2018). These classifications provide a robust framework for analyzing genetic and morphological variation related to major biogeographic units.

### DNA Extraction and Sequencing

DNA was extracted from leg and thorax muscle tissue using a modified saline extraction method (Jowett 1986). We used the DArTseq approach, a modified version of RADseq, that belongs to the broader category of genotyping-by-sequencing (GBS) approaches. DArTseq relies on the use of two restriction enzymes (PstI and SphI) to digest genomic DNA, targeting low-copy regions and enhancing marker density for efficient and cost-effective SNP discovery (Kilian et al. 2012). This protocol was implemented by Diversity Arrays Technology Pty Ltd (Canberra, Australia).

DNA integrity was assessed prior to sequencing through electrophoresis on a 1.2% (w/v) native agarose gel, and DNA concentration was determined using spectrophotometry (Nanodrop 2000, Thermo Fisher Scientific). Only samples with sufficient DNA integrity and quality were selected for sequencing. Specimens from private collections were not used for DNA extraction or sequencing, with the only exception being samples from Isla Robinson Crusoe (Juan Fernández Archipelago), which exhibited low DNA degradation and were processed under the same protocols as field-collected samples.

Digestion of samples (150 ng of DNAg) was performed using a combination of frequent and rare methylation-sensitive restriction enzymes (Pstl and Sphl, respectively) (Kilian et al. 2012). Smaller fragments of the digested DNA (< 200 bp) were ligated to a barcode adapter (6 to 9 bp in length) and amplified by PCR. PCR products were concentration-standardized and pooled for sequencing on a single HiSeq 2500/NovaSeq 6000 lane (Illumina) achieving a read depth of approximately 1.2 million reads per sample (a plate of 94 samples was pooled in each lane).

### Sequence Processing and Alignment

*SNP* calling and sequence processing were performed following DArT's proprietary pipeline (Kilian et al. 2012). Reads were aligned to the *V. cardui* reference genome (NCBI Genome Accession Number: GCF_905220365.1, Scaffold N50: 14.6 Mb, BUSCO Integrity: 99.2%), and variants were filtered based on quality metrics. A sequence quality control step was performed to ensure data accuracy and reliability (Gruber et al. 2024). The data received were analysed using the dartR package (Gruber et al. 2024). A total of 48.872 diploid *SNPs* were obtained for 137 individuals representing 23 localities throughout their distribution range. To ensure high data quality, the dataset was filtered using the gl.filter function, following these criteria: Exclusion of loci with missing data >20%, retention of loci with a genotyping repeatability threshold set to >80% (ensuring high reliability in *SNP* calling), removal of duplicate loci arising from paralogous sequences or sequencing errors (secondary loci), exclusion of monomorphic loci (which lack informative variation), exclusion of individuals with sequence quality <80% (based on overall genotyping success). After applying these filters, 6,027 high-confidence *SNP*s were retained for 136 individuals from 23 localities.

### Genetic Structure, Spatial Patterns, and Genetic Diversity

To establish clusters based on observed genetic diversity, principal coordinates analysis (PCoA) was performed. Subsequently, to assess whether genetic structure was influenced by geographic distance, an Isolation-by-distance (IBD) analysis was carried out. Genetic distance was estimated using the Euclidean method, while geographic distances (in kilometers) between sampling localities were calculated from latitude and longitude coordinates. The relationship between genetic and geographic distance was evaluated using a Mantel test with 999 permutations, as implemented in the R package dartR.spatial (Gruber et al. 2024). In addition, to establish population differentiation or the presence of independent evolutionary lineages, an ancestry analysis was performed by means of an admixture analysis using a sparse non-negative matrix factorization (*sNMF*) approach with a run of 100 replicates performed by the R package LEA (Frichot and François 2015). A number of ancestral populations (*K* = 2) was selected based on cross-validation scores minimizing entropy. Ancestry coefficients were plotted using the barchart function, and the *Q-matrix* values (Caye et al. 2018) were mapped according to the geographical coordinates of the samples in the R package tess3r (Caye et al. 2016), and the graph was edited using the software QGis (QGIS. 2024). Additionally, overall heterozygosity was estimated using the dartR package (Gruber et al. 2024), following the criteria of García-Berro et al. (2023), which suggests that heterozygosity may serve as a proxy for detecting migratory behaviour in butterflies. Species with heterozygosity values ≥2.48 (SE: ± 0.65) are inferred to exhibit migratory behaviour, while species with heterozygosity values close to 1.55 (SE: ± 0.79) are likely sedentary.

To quantify genetic differentiation between the ancestral populations, *F_ST_* was calculated using the gl.fst.pop function from the dartR R package (Mijangos et al. 2022; Gruber et al. 2023). *F_ST_* values were estimated from *SNP*s obtained via DArTseq genotyping, following standard quality control criteria outlined above. Population assignments were determined a posteriori where individuals were assigned to the population corresponding to their highest ancestry proportions in the *Q-matrix* from the LEA-based population structure analysis (Frichot and François 2015). A bootstrap resampling procedure (1,000 replicates) was performed to obtain confidence intervals and assess the statistical significance of the *F_ST_* estimates. The *P-value* was computed as the proportion of bootstrap replicates where *F_ST_* values were greater than or equal to the observed *F_ST_*, testing the null hypothesis of no genetic differentiation between populations.

### Photography and Digitization

To preserve natural wing morphology and minimize distortions, both forewings and hindwings from the same individual were carefully detached and mounted between two glass plates, maintaining their original physiognomic arrangement. Each glass mount was sealed with small adhesive drops at the corners to prevent movement while avoiding pressure on the wings. This method ensured structural integrity and consistency in positioning across samples. Mounted wings were photographed dorsally over millimeter paper using a SONY A6000 camera equipped with a 60 mm macro lens. All photographs were taken under standardized lighting conditions and at a fixed distance to avoid variation in brightness, scale, and perspective. A total of 907 forewings and 866 hindwings were analysed. Due to varying degrees of wing damage, not all individuals had both forewings and hindwings available for analysis. Consequently, the total number of wings does not correspond directly to the number of individuals, and there is no requirement that forewing and hindwing data originate from the same specimen. The number of analysed wings per locality is detailed in supplementary table S1, Supplementary Material online.

**Fig. 2.**
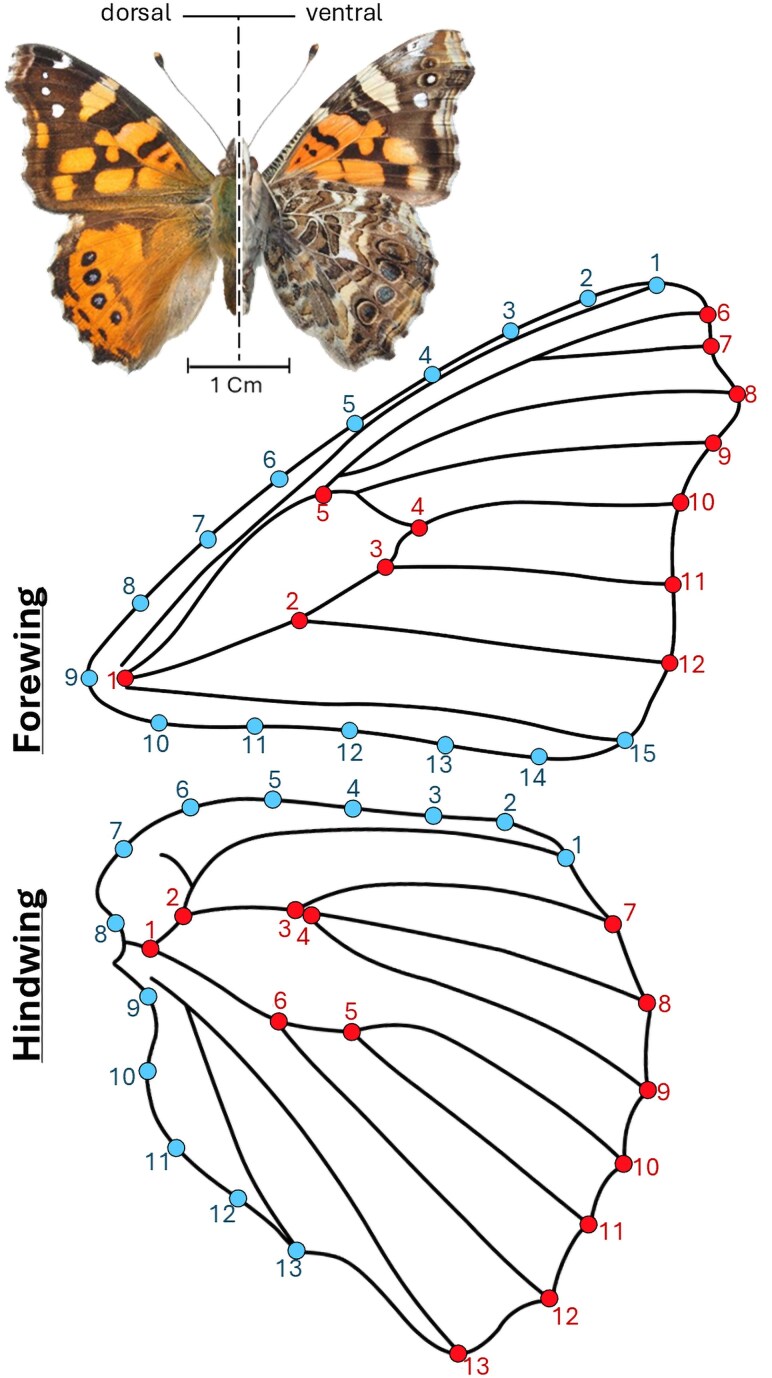
Graphical representation of the forewings and hindwings of *V. carye*, with 12 and 13 landmarks (red), respectively, and with 15 and 13 curve semilandmarks (blue), respectively. The reference image shows the species in dorsal and ventral views, with a scale bar for size reference.

Twelve landmarks and fifteen curve semilandmarks were digitized on each forewing, and thirteen landmarks and thirteen curve semilandmarks on each hindwing, using the R package StereoMorph (Olsen and Westneat 2015). All landmarks were placed at vein intersections or along wing margins (Fig. 2). To ensure consistency and minimize observer bias, all wings were digitized by the same person. Additionally, a second round of digitization was performed for each dataset to assess repeatability (Arnqvist and Martensson 1998; Fruciano 2016).

### Shape Analysis

A Procrustes superimposition was applied to eliminate variation unrelated to shape —translation, rotation, and scaling—prior to analysis (Rohlf and Slice 1990). The 2D coordinates of landmarks were analyzed in MorphoJ v.1.0.7a (Klingenberg 2011) for shape quantification, including centroid size calculation.

To represent the shape space, both PCA and CVA were applied independently to obtain a comprehensive understanding of shape variation and potential group differentiation. A principal component analysis (PCA) was performed using the covariance matrix of Procrustes-aligned coordinates in the R package geomorph (Baken et al. 2021; Adams et al. 2024). PCA allowed exploration of the overall distribution of wing shapes without imposing any a priori grouping, thereby capturing the natural morphological variation across the species’ range. In contrast, a canonical variate analysis (CVA) was conducted using the R package Morpho (Schlager 2017) to enhance morphological differentiation among biogeographic regions, facilitating the detection of spatial patterns. Unlike PCA, CVA does not reflect raw shape variance but instead emphasizes differences among groups, helping reveal ecological or geographic structure. Shape differences were tested with 10,000 permutations, and results were visualized using the ggplot2 package (Wilkinson 2011).

Forewings and hindwings were analysed separately due to their distinct evolutionary origins and ecological functions (Kuussaari et al. 2014; Le Roy et al. 2019).

## Results

### Genetic Clustering and Ancestry Analysis

**Fig. 3.**
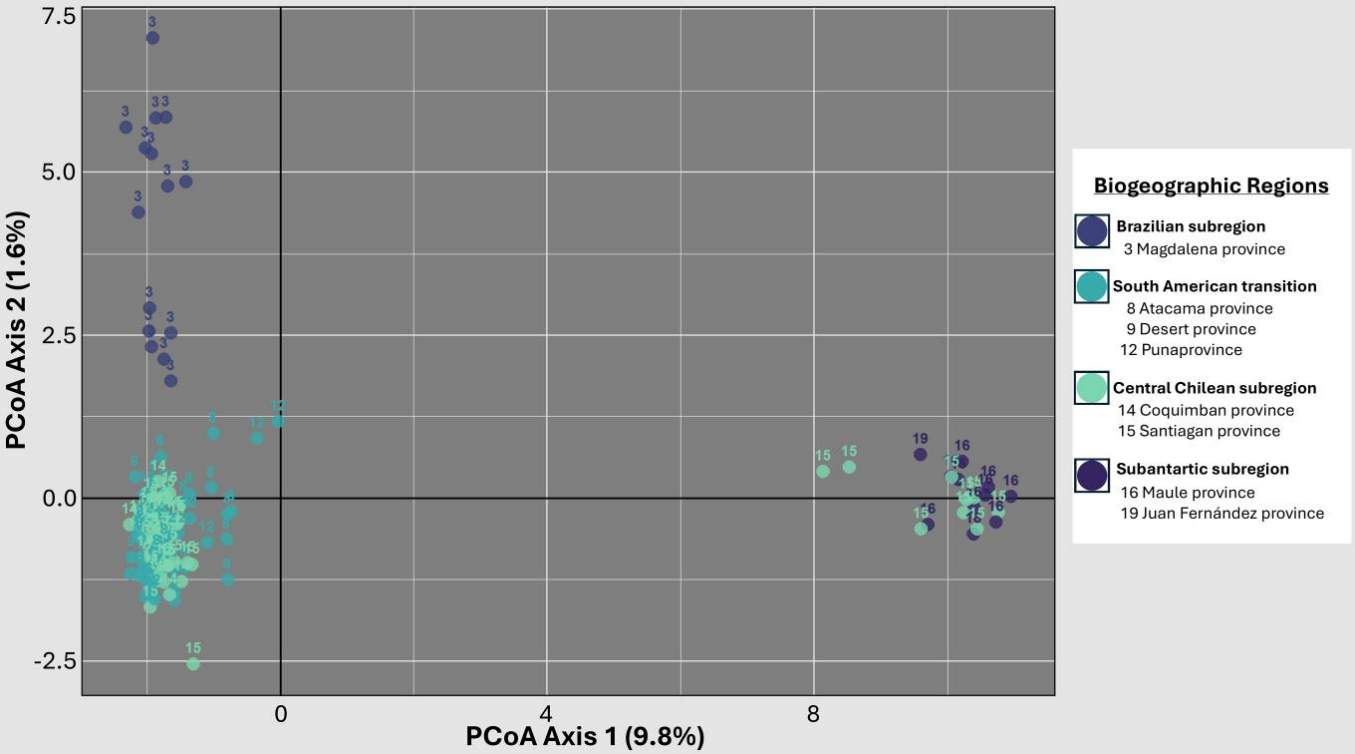
Principal coordinates analyses (PCoA) performed on 6,027 *SNP*s for 136 individuals from 23 localities distributed over ∼5,000 km. The first two coordinates explain 11.4% of the total genetic variation. Points are colored according to their biogeographic region, while the numbers indicate their corresponding biogeographic province (Morrone 2018; Morrone et al. 2022).

**Fig. 4.**
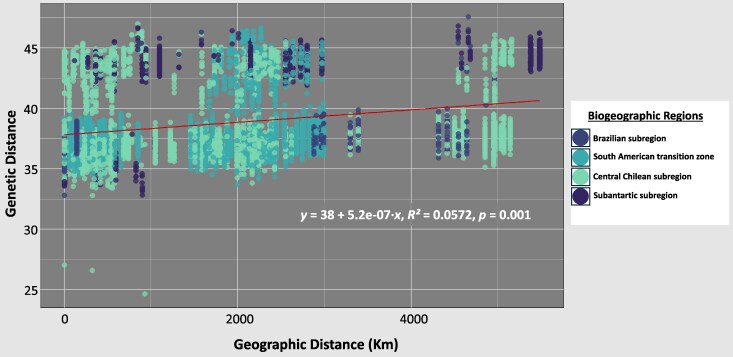
Relationship between geographic distance (Dgeo) and pairwise genetic distance (Dgen) for *V. carye*, based on *SNP* data from individuals collected across its distribution range. Each point represents a pairwise comparison between individuals, and colors indicate the biogeographic region of origin (Morrone 2018; Morrone et al. 2022) for the pair. The regression line illustrates the overall isolation-by-distance (IBD) pattern, with the corresponding equation, coefficient of determination (*R*²), and *P*-value displayed on the plot.

The PCoA analysis (Fig. 3) shows two clusters of individuals, one primarily associated with coastal localities and the other with Andean localities. Additionally, the Mantel test, based on 999 permutations, detected a statistically significant correlation between geographic and genetic distance (*r* = 0.238, *P* = 0.001) (Fig. 4). However, the coefficient of determination (*R^2^* = 0.0572) indicates that geographic distance explains only part of the genetic variability observed, suggesting that factors others than isolation by distance may play a relevant role in shaping the genetic structure of *V. carye.*

**Fig. 5.**
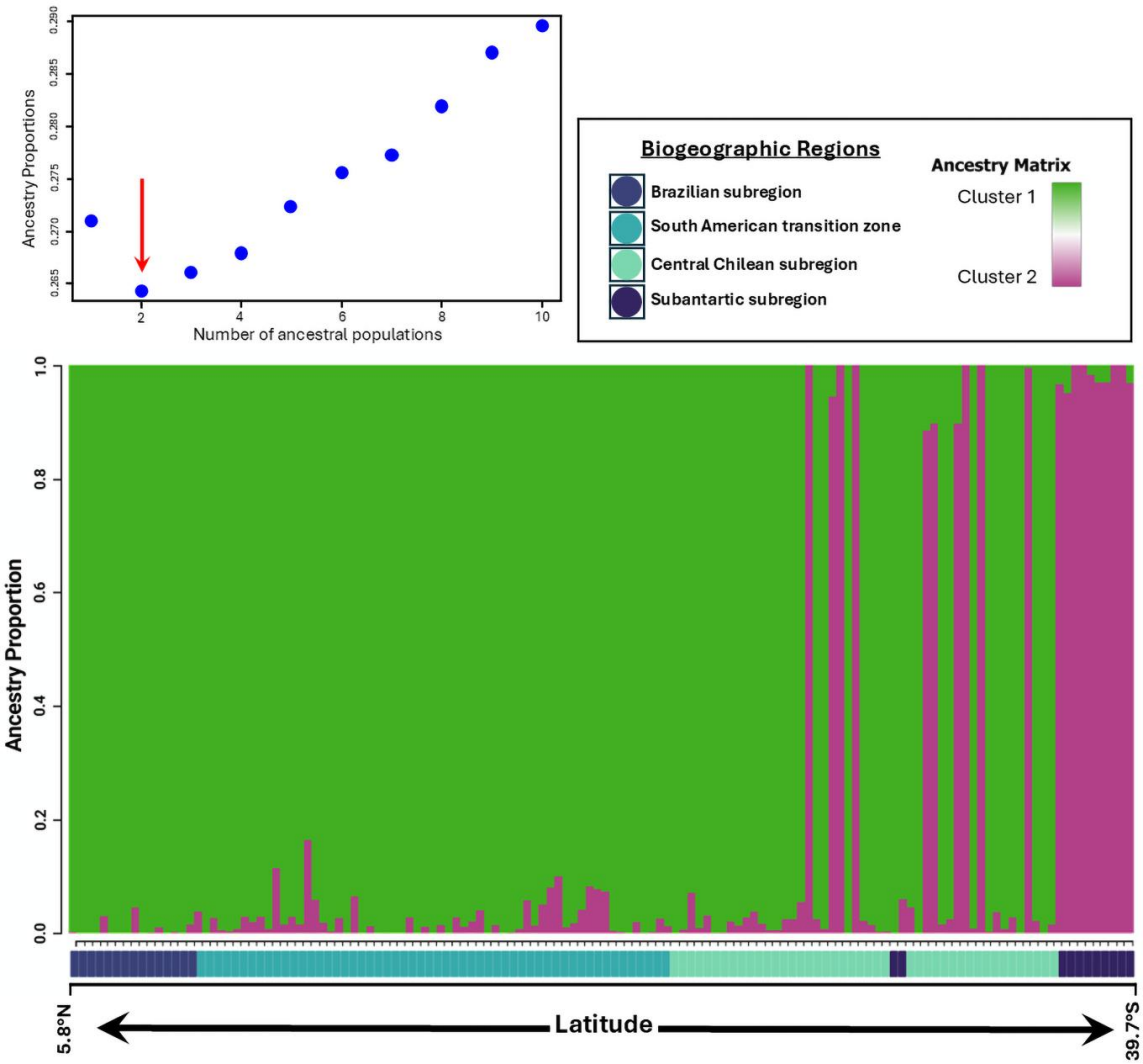
Ancestry analysis of *V. carye* based on ancestry coefficients for *K* = 2 ancestral populations. Each vertical bar represents an individual, and the color indicates the proportion of ancestry from each genetic cluster. Individuals are ordered from north to south according to their latitude, and their corresponding biogeographic regions are displayed below the plot (Morrone 2018; Morrone et al. 2022). The inset graph shows Cross-entropy values for each *snmf* run with *K* ancestral populations ranging from 1 and 10, with a red arrow indicating the most probable *K* value.

The ancestry analysis (Fig. 5) suggests the presence of two main genetic clusters, one associated with the Andes (green) and another with the Pacific Ocean coast (magenta). However, there is no evidence of strong genetic structure between these clusters, as most localities contain individuals with varying proportions of both ancestries. The presence of individuals with mixed ancestry across the entire range indicates that these clusters do not represent fully structured populations or independent evolutionary lineages; rather, they reflect historical patterns of gene flow and population connectivity. This is further supported by the low level of genetic differentiation between clusters (F_ST_ = 0.233, *P* = 0.497), indicating no statistically significant genetic divergence. These results suggest that the observed clustering pattern primarily reflects shared ancestry rather than reproductive isolation or restricted gene flow.

**Fig. 6.**
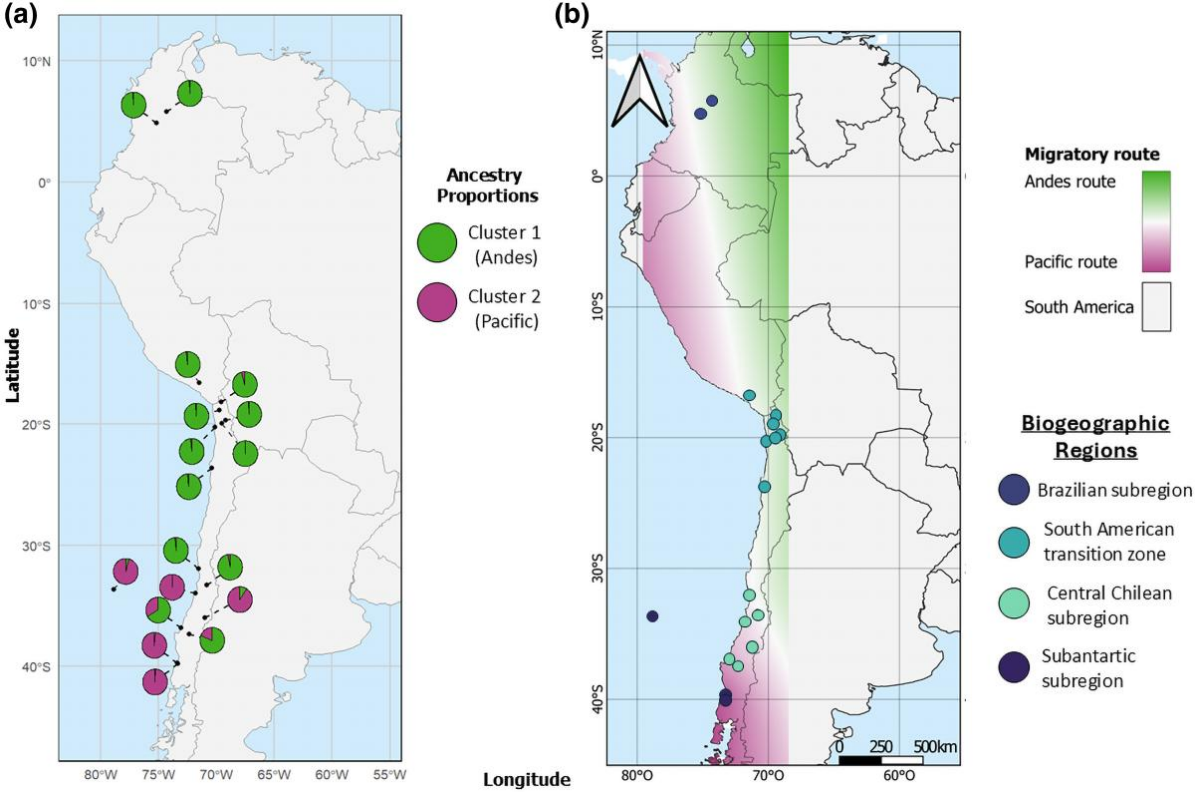
a) Ancestry proportions of *V. carye* at each sampled locality, inferred from *snmf* analyses. Pie charts show the average proportion of each genetic cluster per locality: Cluster 1 (green) and Cluster 2 (magenta). b) Spatial gradients representing the probability of occurrence for each of the two inferred ancestral populations (*K* = 2), derived from ancestry matrix values. These gradients suggest two distinct migratory routes across South America: an Andean route (green) and a Pacific route (magenta). Sampled localities are indicated by points colored according to their biogeographic subregion (Morrone 2018; Morrone et al. 2022).

Likewise, the representation of ancestry values in the geographical matrix (Fig. 6) indicates a population with a probable coastal distribution and another with a probable Andean distribution (colors on the map correspond to those in the ancestry barplot).

Finally, the average heterozygosity (Ho) for the 136 specimens of *V. carye* was 5.74% (4.7% to 8.8%) (SE: ± 0.048%), as detailed in supplementary table S2, Supplementary Material online.

### Shape Variability

**Fig. 7.**
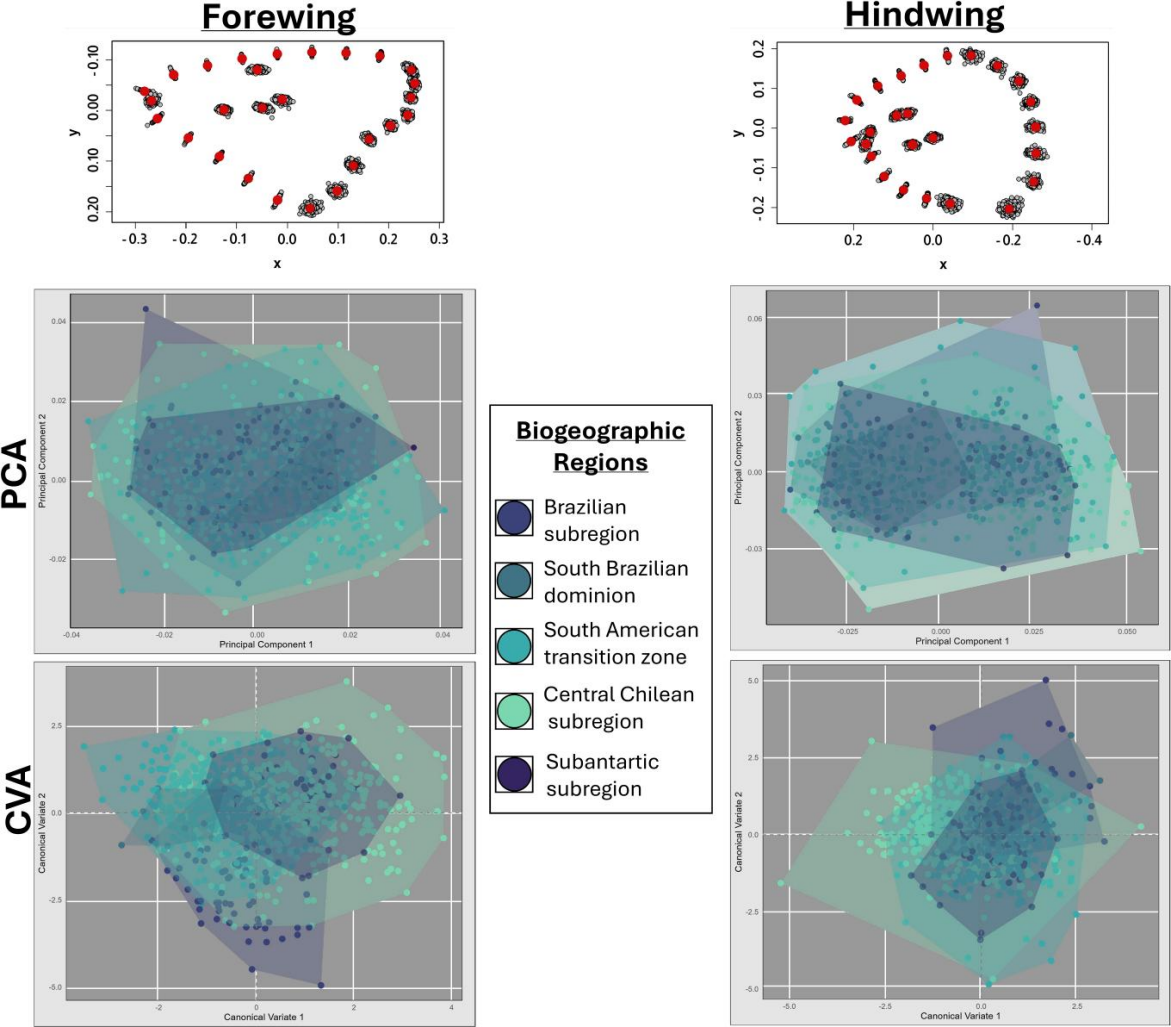
Principal component analysis (PCA) and canonical variate analysis (CVA) of *V. carye* wing shape variation. The top panels display the mean shape and deformation grids for the forewings (left) and hindwings (right). The middle panels show the PCA results, while the bottom panels display the CVA. Individuals are colored according to their corresponding biogeographic region (Morrone 2018; Morrone et al. 2022), and polygons represent the morphospace occupied by each region.

**Fig. 8.**
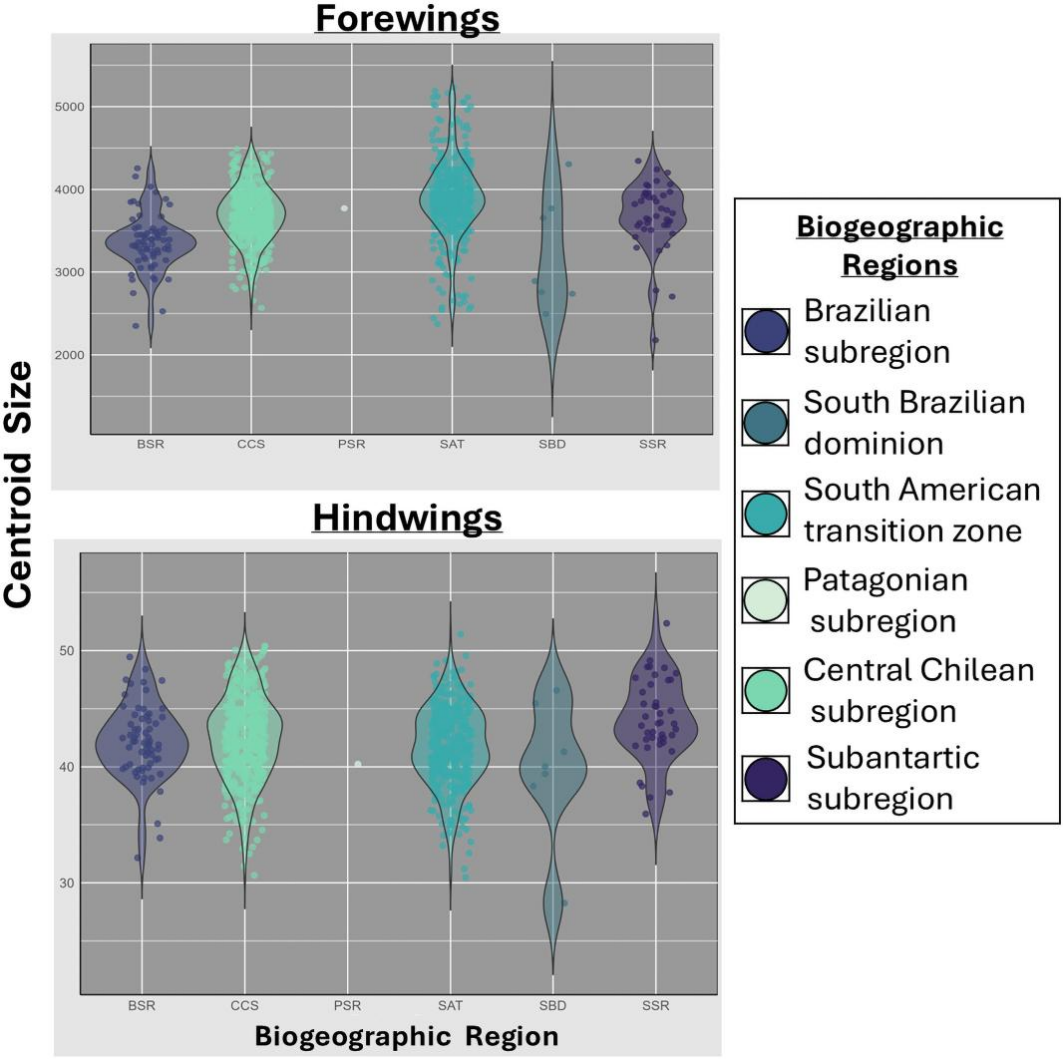
Violin plots depicting the variation in centroid size for forewings (top) and hindwings (bottom) of *V. carye*. Each point represents an individual, and colors correspond to the biogeographic regions (Morrone 2018; Morrone et al. 2022): Brazilian subregion (BSR), Central Chilean subregion (CCS), Patagonian subregion (PSR), South American transition zone (SAT), South Brazilian dominion (SBD), and Subantarctic subregion (SSR). The width of each violin plot illustrates the density distribution of centroid size values within each biogeographic region.

The PCA analysis performed between the first two principal components (Fig. 7, middle panels), which accounts for 60.8% of forewing shape variation and 63.4% for hindwing shape, reveals an absence of shape differentiation between localities distributed along the latitudinal gradient of the species’ distribution. These results are consistent with the CVA (Fig. 7, bottom panels), which, although this analysis emphasizes group differentiation, the observed differences were not statistically significant (Campbell and Atchley 1981). Finally, the centroid size analysis (Fig. 8) shows a complete lack of size variation across the species’ range.

## Discussion

*SNP* analyses reveal weak genetic structure throughout the species’ range, with two broadly distributed genetic clusters and low differentiation between them. This pattern suggests that *V. carye* does not correspond to a complex of cryptic species. The ancestry analysis shows the presence of two populations distributed in parallel along ∼5,000 km between Los Nogales in Colombia and Valdivia in Chile, following the Pacific Ocean and the Andes. Although most individuals are predominantly assigned to one of the clusters, the low *F_st_* values, the absence of a significant IBD pattern, and the high levels of observed heterozygosity collectively are consistent with high dispersal potential and patterns of widespread gene flow. Furthermore, the presence of individuals with mixed ancestry between both populations indicates historical or recent connectivity. Accurately determining the timing and extent of gene flow would require further analyses using demographic modeling approaches. One potential limitation of our dataset is the presence of a sampling gap between ∼4°S and ∼16°S, where no genomic data were available. While this could theoretically introduce a bias in the inference of genetic connectivity, the assumptions of the clustering algorithms used for ancestry estimation (LEA) and spatial population structure inference (TESS3r) suggest that this should not significantly affect the results. Specifically, these methods are designed to detect genetic discontinuities even in the presence of gaps in geographic sampling, allowing for a robust estimation of population structure. Therefore, while finer-scale genomic sampling in this region could provide additional resolution, the overall lack of strong genetic structure and the continuous admixture patterns observed suggest a high degree of genetic connectivity throughout the species’ range, even across this unsampled genomic region. This interpretation is further supported by the pattern observed in the IBD plot (Fig. 4). Rather than showing a gradual increase in genetic divergence with geographic distance—as expected under the stepping-stone model—the data reveal considerable overlap in genetic distances across the entire ∼5,000 Km gradient. Genetic divergence between distant individuals is not consistently higher than that observed among geographically closer ones, and the regression line indicates only a weak association (*R*^2^ = 0.0572, *P* = 0.001). The two bands of points observed likely reflect the two ancestry components inferred by the admixture analysis. While the absence of samples between ∼4°S and ∼16°S might contribute to the discontinuity in the plot, the fact that the densest concentration of pairwise comparisons occurs between Colombian and Peruvian samples suggests that this gap is not solely responsible. Instead, the parallel distribution of these clusters across the geographic axis, coupled with low overall divergence, reinforces the conclusion that geographic distance has limited influence on genetic differentiation in *V. carye*, consistent with high dispersal potential and widespread genetic connectivity.

The findings are further supported by the low level of genetic differentiation between the two clusters (*F_ST_* = 0.233, *P* = 0.497), indicating no statistically significant genetic divergence. The observed lack of strong genetic structure is consistent with species exhibiting high dispersal potential, where connectivity between populations is maintained despite geographic distance. These results are concordant with those observed in the well-documented migratory species *D. plexippus* (Altizer and Davis 2010), where two large populations corresponding to distinct migratory routes exhibit continuous gene flow, a pattern commonly observed in migratory organisms (Liedvogel et al. 2011; Kim and Sappington 2013; Calderón et al. 2016; Lukhtanov et al. 2016; Reppert et al. 2016; Alvial et al. 2017; Battey and Klicka 2017; Merlin and Liedvogel 2019; Talla et al. 2020; García-Berro et al. 2023). Because migratory route fidelity is a key trait subject to selection in long-distance travelers (Dingle and Drake 2007; Ramenofsky and Wingfield 2007; Seeb et al. 2011b; Dingle 2014; Micheletti et al. 2018; Somveille et al. 2021), if genetic structuring were to occur in a species with high movement capability, it would likely reflect differentiation between migratory routes rather than geographic barriers (Miller-butterworth et al. 2003; Ruegg et al. 2014; Battey and Klicka 2017; Álvarez-Varas et al. 2021; Bay et al. 2021; Kersten et al. 2021). Similarly, gene flow between migratory routes has been extensively documented in other species with migratory behaviour (Dockx 2007; Liedvogel et al. 2011; Kim and Sappington 2013; Pfeiler and Markow 2017; Alvial et al. 2019; Talla et al. 2020). Our findings also align with García-Berro et al. (2023), who proposed that species with genome-wide heterozygosity values >2.48% are likely migratory, while those closer to 1.55% tend to be sedentary. In our study, observed heterozygosity values ranged from 4.7% to 8.4% (mean = 5.74%, SE = ±0.048%) based on 48,872 *SNPs* from 136 individuals sampled over ∼5,000 Km. These values exceed the threshold for migratory species and provide further support for high genetic connectivity across species’ range. While high heterozygosity can also be associated with large population size or ecological generalism, its co-occurrence with morphological and genetic homogeneity reinforces the interpretation of *V. carye* as a species with high dispersal potential, consistent with a migratory lifestyle.

Based on the geographical distribution of ancestry proportions, our findings support the existence of two migratory routes: one associated with the Pacific Ocean and another with the Andes. These routes appear to maintain constant genetic connectivity along the species’ range. The spatial signal of these clusters is visualized in Fig. 6b as color gradients representing the probability of geographic occurrence for each of the two inferred ancestral populations (*K* = 2), suggesting the existence of structured movement pathways. Cluster 1 (green) corresponds primarily to the Andean migratory route, while Cluster 2 (magenta) is associated with the Pacific coastal route. Although the extremes of the distribution are predominantly composed of one of the two clusters, a few localities—including Los Nogales in Colombia—show low levels of admixture, which may reflect occasional gene flow or secondary contact. Further sampling in the intermediate range would help clarify whether these corridors converge at specific breeding areas or whether admixture is more widespread than currently detected. It is also important to highlight that the samples from the Juan Fernández Archipelago in the admixture analysis appear grouped with coastal localities, which reinforces the hypothesis of a Pacific-associated route. This is in accordance with Wahlberg and Rubinoff (2011), who proposed that *V. carye* exhibits high movement capability, using the distance between its insular and continental localities as a proxy. It also aligns with observations in *V. cardui*, another *Vanessa* species well-studied for its migratory behaviour. In that case, individuals have been observed performing trans-oceanic flight of over 4,200 Km from West Africa to French Guiana in South America (Suchan et al. 2024), without showing genetic differences between long- and short-distance migrants (Reich et al. 2025). Our results similarly show gene flow between individuals collected on the Juan Fernández Archipelago (>670 km from the mainland) and those collected in both the northernmost (Los Nogales and Murillo, Colombia) and southernmost parts of the species’ distribution.

This pattern resembles that observed in other widely distributed species without migratory behaviour. However, these species differ from *V. carye* mainly due to a stepping-stone pattern, in which geographically closer populations are genetically more similar to each other. For example, *Leopardus geoffroyi* shows two genetic clusters with gene flow between them, interpreted as the result of two recent bottleneck events—one caused by habitat loss from deforestation during World War I in the Argentine Amazon, followed by recovery and secondary contact (Bou et al. 2021). A similar case occurs in *Zonotrichia capensis*, which is distributed from Chiapas in Mexico to Tierra del Fuego in Chile and Argentina. Despite some behavioral differences and the existence of geographically restricted populations, gene flow has been maintained across its range due to population proximity, preventing speciation (Van Dongen et al. 2010; Lougheed et al. 2013; Campagna et al. 2014). In contrast, in *V. carye*, the putative populations occur in parallel across several thousand kilometres, suggesting a different kind of structuring more compatible with a migratory or highly dispersive species.

Regarding wing shape variability, our results indicate a lack of significant morphological differences across the species’ range. This suggests that its wing morphology in *V. carye* is conserved despite the broad environmental gradient it inhabits, providing no support for local morphological adaptation. In butterflies, wing shape is strongly shaped by ecological and functional pressures and often reflects habitat-specific adaptations (Chazot et al. 2016; Le Roy et al. 2019). For instance, in the genus *Morpho*, wing shape varies with vertical habitat use, with canopy individuals showing distinct shapes compared to understory individuals (DeVries et al. 2010). Similarly, *Heliconius* species show shape variation in response to habitat physicochemical features, supporting the role of local adaptation (Dell’Aglio et al. 2022). However, in *V. carye*, the lack of differentiation suggests an optimized morphology for broad dispersal rather than local adaptation. The PCA analyses revealed that 60.8% and 63.4% of the total shape variation for the forewings and hindwings, respectively, are captured in the first principal component (PC1). This indicates that most of the morphological variation is concentrated along a single axis, making PC1 a useful and informative variable for detecting potential patterns of morphological differentiation. However, no evident patterns or clustering were detected along the analyses, supporting the hypothesis of conserved morphology across the range. The CVA analysis also supported overall morphological consistency, with no distinct clusters across the ∼7,000 km latitudinal gradient. Our findings are consistent with Escobar-Suárez et al. (2023), who found no significant variation in *V. carye* wing morphology across its altitudinal gradients, further supporting a conserved morphology associated with high mobility. While a wing shape differentiation was expected along such a wide environmental range, our results indicate that *V. carye* possesses an optimized wing morphology for sustained flight throughout its distribution. This morphological uniformity, combined with the genetic evidence of homogeneity, reinforces the hypothesis of high movement capability and supports the notion of migratory behaviour in this species.

Studies on *D. plexippus* have shown wing shape variation between populations with distinct migratory strategies: Migratory populations tend to have longer and more pointed wings, while nonmigratory populations exhibit shorter and rounder wings. Even among migratory populations, those traveling longer distances show more pronounced aerodynamic traits (Dockx 2007; Altizer and Davis 2010; Li et al. 2016). However, Berns (2014) found that variation in *D. plexippus* can also reflect factors like sex and larval host plant chemistry, indicating that other environmental pressures can influence wing shape even in migratory species. In *V. carye*, observed shape variability does not align with the assigned biogeographic ecoregions and may instead reflect differences due to sex or larval diet. While sexual dimorphism is generally not evident in migratory butterflies (Dingle 2014), some differences related to courtship or oviposition behaviour have been reported (Scott 1975; Scalco et al. 2016). To confirm sex-based variation, genitalia dissection is needed (Sánchez 2008). As for larval feeding, it is important to consider that collection site may not reflect the developmental site in migratory species. Controlled rearing and common garden experiments would be required to disentangle these potential sources of morphological variation (Berns 2014; Chowdhury et al. 2022).

Although direct individual-level evidence of movement capability is still needed for *V. carye*, our molecular and morphological results suggest high dispersal capacity. Genetic analyses indicate two broadly distributed ancestral populations with low levels of genetic differentiation across ∼5,000 km, and wing shape remains conserved along this entire range. These findings support the hypothesis that *V. carye* constitutes a single evolutionary unit, rather than a cryptic species complex. The combination of low genetic structure, continuous admixture, and morphological uniformity is consistent with species exhibiting high mobility and provides a robust framework for future research into the movement ecology of *V. carye*.

## Supplementary Material

msaf212_Supplementary_Data

## Data Availability

The data underlying this article are available in its online supplementary material, Supplementary Material online.
